# Supplementary material for: Netcombin: An algorithm for constructing optimal phylogenetic network from rooted triplets
Source: PLoS One. 2020 Sep 18;15(9):e0227842. doi: 10.1371/journal.pone.0227842 (PMC7500971; doi:10.1371/journal.pone.0227842)
Supplement: S4 File — (ZIP) [file pone.0227842.s004.zip › SequenceData-Triplet/sequenceData-10-20-30-0-Triplet/length 300, numseq 40, rate 10^-7/4 seq.pdf]

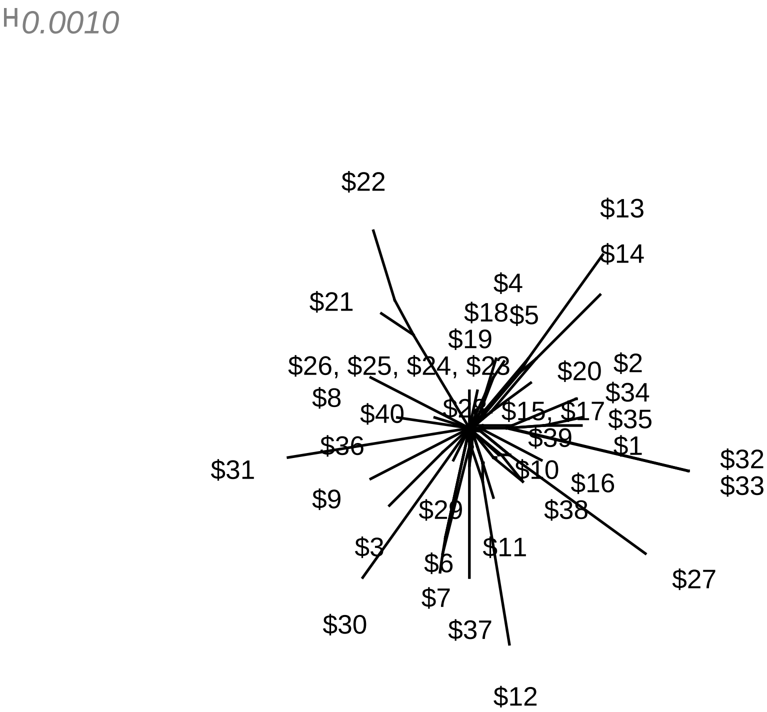
0.0010
$22
$13
$14
$4
$21
$18
$5
$19
$26, $25, $24, $23
$20
$2
$34
$8
$40
$15, $17
$35
$39
$36
$1
$31
$32
$10
$33
$16
$9
$29
$38
$3
$11
$6
$27
$7
$30
$37
$12
